# Lower Body Perfusion Reduces the Morbidity of Postoperative Acute Kidney Injury in Type A Dissection: A Propensity-Matched Analysis

**DOI:** 10.21470/1678-9741-2022-0190

**Published:** 2023

**Authors:** Zhuo Tang, Ying Lv, Bin Wang, Zhonglu Yang, Yu Liu, Hui Jiang

**Affiliations:** 1 Graduate School, China Medical University, Shenyang, People’s Republic of China; 2 Department of Cardiovascular Surgery, Second Affiliated Hospital, Zhejiang University School of Medicine, Hangzhou, People’s Republic of China; 3 Department of Cardiovascular Surgery, General Hospital of Northern Theater Command, Liaoning, People’s Republic of China

**Keywords:** Acute Kidney Injury, Aneurysm, Dissecting, Aorta, Thoracic, Cardiopulmonary Bypass, Constriction, Temperature, Drainage

## Abstract

**Introduction:**

Lower body perfusion (LBP) is a technique used to provide blood perfusion to distal organs and spinal cord during circulatory arrest. However, the effect of LBP on the prognosis of aortic arch surgery, especially on postoperative renal function, remains unclear.

**Methods:**

A total of 304 patients with acute type A aortic dissection who underwent total aortic arch replacement combined with frozen elephant trunk implantation between May 2016 and December 2021 were retrospectively analyzed. The patients were divided into LBP group (group L, n=85) and non-LBP group (group NL, n=219). Routine lower body circulatory arrest was applied during operation in group NL, and antegrade LBP combined was applied during operation in group L. Perioperative data were recorded. Propensity score matching was used for statistical analysis.

**Results:**

After propensity score matching, 85 pairs of patients were successfully matched. Two groups significantly differed in circulatory arrest time (six minutes vs. 30 minutes, P=0.000), cross-clamping time (101 minutes vs. 92 minutes, P=0.010), minimum nasopharyngeal temperature (29.4ºC vs. 27.2ºC, P=0.000), and highest lactate value during cardiopulmonary bypass (2.3 µmol/L vs. 4.1 µmol/L, P=0.000). Considering the postoperative indicators, the drainage volume (450 mL vs. 775 mL, P=0.000) and the incidence of level I acute kidney injury (23.5% vs. 32%, P=0.046) in group L was lower than those in group NL.

**Conclusion:**

LBP resulted as a safe and feasible approach in aortic arch surgery, as it could significantly shorten the circulatory arrest time, which might reduce the incidence of postoperative level I acute kidney injury.

## INTRODUCTION

Acute aortic dissection is a life-threatening aortic disease with high incidence and mortality^[1]^. In recent years, moderate hypothermic circulatory arrest (MoHCA) combined with selective antegrade cerebral perfusion (SACP) has been widely used during acute aortic dissection, proving to be a safe and effective approach^[2]^. Previous studies found that the nervous system injury caused by cardiopulmonary bypass (CPB) was related to the rewarming process and cerebrovascular autoregulation dysfunction, while excessive rewarming rate was positively correlated with aggravation of nerve injury. As the body temperature in total arch replacement surgery has an increasing trend, the ischemic tolerance of the spinal cord and viscera during circulatory arrest at higher temperature has gained increasing interest.

Lower body perfusion (LBP) is a technique used for the provision of blood perfusion to distal organs and spinal cord during traditional circulatory arrest. This approach can solve the problem of intraoperative distal organ ischemia and significantly shorten the circulatory arrest time, thus making it possible to raise the temperature during circulatory arrest in aortic arch surgery^[3]^. Our previous studies found that applying antegrade LBP can significantly shorten the circulatory arrest time to 10 (8,11) minutes, thus significantly shortening the ischemia and hypoxia time of visceral organs and spinal cord^[4]^. In addition, cardiac surgery-associated acute kidney injury (CSA-AKI), which commonly occurs after aortic arch surgery, can seriously affect the short-term and long-term prognoses of patients^[5]^. Kidney ischemia and hypoxia during CPB, especially during circulatory arrest, are important risk factors for acute kidney injury (AKI) after aortic arch surgery. In patients undergoing emergency surgery for acute type A aortic dissection, the incidence of postoperative AKI has great fluctuations, ranging between 20.2% and 66.7%^[6]^. However, it is still unclear whether LBP can reduce the incidence of postoperative AKI.

This study retrospectively analyzed and summarized the changes of antegrade LBP on CPB-related factors in aortic arch surgery so as to evaluate the impact of antegrade LBP on the prognosis of aortic arch surgery, especially on postoperative renal function.

## METHODS

### Clinical Information

A total of 304 patients with acute type A aortic dissection who underwent total aortic arch replacement combined with frozen elephant trunk implantation in our department between May 2016 and December 2021 were included in this retrospective analysis. All patients were diagnosed with Stanford type A aortic dissection by computed tomography angiography before surgery. According to whether LBP was applied during the operation, 85 patients were assigned to the LBP group (group L) and 219 patients to the non-LBP group (group NL). All operations were performed by the same group, including surgeons, anesthesiologists, perfusionists, cardiologists, and nurses. Inclusion criterion was the following: acute Stanford type A aortic dissection affecting the aortic arch. Exclusion criteria were: (1) neurological complications, including intracerebral hemorrhage and cerebral infarction; (2) hypoperfusion syndrome; (3) patients treated with renal transplantation and maintenance dialysis; and (4) concomitant surgery requiring total sternotomy (coronary heart disease, mitral valve disease, and congenital heart disease). General preoperative information and surgery-related data were collected, and clinical endpoints were recorded.

This study was approved by the ethics committee of our hospital (ethical Review K [2020] 19). All patients signed informed consent.

### Surgical Methods and Management of Cardiopulmonary Bypass

All patients received venous inhalation anesthesia. After endotracheal intubation, the left radial artery and the right dorsalis pedis artery were punctured to monitor the blood pressure of the upper and lower limbs. The pulmonary artery floating catheter (Swan-Ganz catheter) was then implanted through the internal jugular vein. Cerebral protection was performed with an ice bag under the head. Surgical operation was performed as previously described^[7]^. Disinfection and sheet laying were performed. A longitudinal skin incision from the superior sternal fossa to the level of the fourth intercostal was made with a length of about 10-15 cm, and the sternum was cut horizontally to the right from the sternal notch to the fourth intercostal space in a “J” shape. The brachiocephalic trunk, the left common carotid artery, and the left subclavian artery were separated. CPB was established by intubation of the innominate artery (right subclavian artery, right common carotid artery, or left common carotid artery) as arterial perfusion tube, secondary intubation of the right atrial vena cava as venous drainage tube, and intubation of the right superior pulmonary vein as left atrial drainage tube. After initiation of CPB, the temperature gradually decreased. After clamping and transection of the aorta, 4:1 cold-blooded myocardial protection solution was directly perfused through the openings of the left and right coronary arteries for myocardial protection. After cardiac arrest, the aortic root was treated according to the results of preoperative ultrasound and intraoperative exploration, including Bentall operation, aortic valvuloplasty, and/or aortic valve replacement.

**Group NL**: the routine lower body circulatory arrest strategy was employed. When the nasopharynx temperature dropped to 25ºC-27ºC, and the anus temperature dropped to 28ºC-30ºC, the left common carotid artery, left subclavian artery, and innominate artery were occluded, and the lower body circulation was arrested. The 15-Fr femoral artery cannula was inserted into the left common carotid artery (or right common carotid artery). The innominate artery cannula and femoral artery cannula were connected by the “Y”-shape connecting tube through the myocardial protection perfusion pump. Bilateral SACP was performed with the perfusion flow maintained at 3-5 mL/kg/min. The nerve system injury was monitored by near-infrared spectroscopy, after which the left common carotid artery, left subclavian artery, and innominate artery were cut off, and the aorta was occluded at the distal end of the left subclavian artery. A 26-28-cm elephant trunk stent was then implanted into the end of the descending aorta with the air bag inflated. The descending aorta was anastomosed and fixed with four branches of artificial blood vessels, and lower body circulation was restored after exhausting through the distal branch of artificial blood vessels. Next, the artificial vessel branch was end-to-end anastomosed with the left common carotid artery. After anastomosis, the bilateral cerebral perfusion was restored, and the temperature was slowly raised through CPB. At this time, the proximal end of the ascending aorta was end-to-end anastomosed with the artificial vessel. After anastomosis, the aorta was opened to restore blood supply of the coronary artery. Finally, the remaining two branches of artificial blood vessels were end-to-end anastomosed with the innominate artery and left subclavian artery^[7]^.

**Group L**: antegrade LBP combined with a short-term circulatory arrest strategy was performed following the same approach used in the previous study^[4]^, which could briefly be explained as follows: when the nasopharynx temperature dropped to 30ºC-32ºC, and the anus temperature dropped to 32ºC-34ºC, the three branches of the aortic arch were occluded, and lower body circulation was arrested. Bilateral SACP was performed. Nerve system injury was monitored by near-infrared spectroscopy during the operation. The innominate artery was clamped, circulation was arrested, and frozen elephant trunk stent was implanted. After implanting the elephant trunk stent into the distal end of the descending aorta, 16-Fr vena cava cannula with a balloon was used to occlude the four branches of artificial blood vessels, and antegrade LBP was performed with a flow rate of 25 mL/kg/min. Next, four branches of artificial blood vessels were anastomosed with an elephant trunk stent. After anastomosis, antegrade LBP was converted from vena cava intubation with a balloon to four branches of artificial blood vessels for perfusion. According to the conventional operation process of total arch replacement, the anastomosis of the left common carotid artery, the proximal anastomosis of the ascending aorta combined with the opening of the ascending aorta to restore the blood supply to the heart, and the anastomosis of the left subclavian artery and innominate artery were successively completed.

### Definition of Complications

According to the Kidney Disease Improving Global Outcomes diagnostic criteria^[8]^, the postoperative AKI diagnostic and grading criteria are shown in Table 1. Perioperative blood transfusion was defined as an intraoperative and postoperative infusion of red blood cells, fresh frozen plasma, and platelets.

**Table 1:** Acute kidney injury classification.

Level	sCr	Urine volume
Level I	sCr increased ≥ 0.3 mg/dl within 48 h (≥ 26.4 µmol/L) or sCr increased by 150%~200% (1.5~2.0 times) the baseline level	Continued 6~12 h of urine volume < 0.5 mL/kg/h
Level II	sCr increased by 200%~300% (2.0~3.0 times) the baseline level	Continued 12 h of urine volume < 0.5 mL/kg/h
Level III	sCr increased ≥ 300% (≥ 3.0 times) the baseline level or sCr ≥ 4.0 mg/dl (≥ 354 µmol/L) or hemofiltration treatment was needed or glomerular filtration rate of patients < 18 years old decreased to < 35 mL/(min·1.73 m^2^)	Continued 24 h of urine volume < 0.3 mL/kg/h or no urine for 12 h

sCr=serum creatinine

### Statistical Analysis

R software was used for statistical analysis. The measurement data with normal distribution were represented as mean ± standard deviation. The independent *t*-test was used for the comparison. Data with non-normal distribution were represented by median and interquartile spacing *(P25, P75)*. Mann-Whitney U test was performed for the comparison. The counting data were represented as the number of cases (%). χ^^2^^ test or Fisher’s exact test was performed for the analysis. The patients were divided into group L and group NL based on the application of LBP during the operation. *P*<0.05 was statistically significant. Propensity score matching (PSM) was conducted between the two groups to simulate randomization in this observational study. PSM was estimated by logistic model and matched between the two groups with optimal model. The covariates were based on the results of standardized mean difference (SMD), of which SMD > 0.1 was enrolled as covariates.

## RESULTS

### Comparison Between the Two Groups Before Matching

The information of patients from the two groups before matching is shown in Table 2. Among the 12 covariates before matching, smoking history (53 [62.4] *vs.* 109 [49.8], *P*=0.048) and aortic valvuloplasty (37 [43.5] *vs.* 68 [31.1], *P*=0.040) were unevenly distributed between the two groups. The differences in intraoperative circulatory arrest time, cross-clamping time, minimum nasopharyngeal temperature, and the incidence of perioperative blood transfusion were all statistically significant (all *P*<0.05). The differences in postoperative drainage volume, ventilator time, maximum lactate value, and the incidence of level II AKI were also statistically significant (all *P*<0.05). Smoking history, diabetes mellitus, hypertension, hyperlipidemia, age, ejection fraction, and aortic valvuloplasty were used as covariates for PSM with SMD > 0.1. Finally, 85 pairs were successfully matched. The baseline characteristics of the information in the two groups were comparable after matching, as shown in Table 3.

**Table 2:** Comparison of perioperative influencing factors before matching.

Indicator	Group L^*^ (n=85)	Group NL^*^ (n=219)	*P*-value	SMD
Demographic characteristics				
Male (%)	65 (76.5)	162 (74.0)	0.653	0.058
Age, years (X±s)	52.0±11.0	50.4±11.1	0.258	0.145
Weight (kg)	76.9±14.3	78.3±14.9	0.469	0.094
Preoperative factors				
Smoking history (%)	53 (62.4)	109 (49.8)	0.048	0.256
Diabetes (%)	1 (1.2)	9 (4.1)	0.198	0.184
Hypertension (%)	70 (82.4)	170 (77.6)	0.364	0.118
Hyperlipidemia (%)	8 (9.4)	9 (4.1)	0.071	0.212
Preoperative Cr (µmol/L)	66.8 (58.2, 96.1)	71.0 (61.0, 97.3)	0.371	0.057
LVEF (X±s)	57.7±2.7	57.4±3.0	0.424	0.105
Perioperative factors				
Aortic valvuloplasty (%)	37 (43.5)	68 (31.1)	0.040	0.26
Aortic valve replacement (%)	3 (3.5)	6 (2.7)	0.715	0.045
Bentall^**^ (%)	11 (12.9)	35 (16.0)	0.507	0.087
CPB time (min)	168 (146, 199)	166 (149, 191)	0.665	
Circulatory arrest time (min)	6 (5, 10)	31 (21, 38)	0.000	
Cross-clamping time (min)	101 (85, 127)	96 (82, 113)	0.025	
Minimum nasopharyngeal temperature (ºC)	29.4 (28.8, 30.4)	26.6 (25.0, 27.9)	0.000	
Perioperative blood transfusion (%)	52 (61.2)	177 (80.8)	0.000	
Maximum lactate during CPB (µmol/L)	2.3 (1.9, 3.2)	4.7 (3.9, 5.1)	0.000	
Postoperative factors				
Second operation (%)	2 (2.4)	5 (2.3)	0.971	
Second intubation (%)	4 (4.7)	6 (2.7)	0.388	
Drainage volume (mL)	450 (330, 650)	780 (505, 1130)	0.000	
Ventilator time (h)	32 (19, 53)	22 (17, 44)	0.046	
ICU time (h)	45 (42, 88)	43 (35, 87)	0.150	
Postoperative hospital stay (days)	15 (11, 21)	15 (11, 20)	0.890	
Death (%)	7 (8.2)	14 (6.4)	0.570	
Postoperative AKI				
AKI (%)	42 (49.4)	115 (52.5)	0.627	
Level I AKI (%)	20 (23.5)	76 (34.7)	0.060	
Level II AKI (%)	19 (22.4)	27 (12.3)	0.029	
Level III AKI (%)	6 (7.1)	12 (5.5)	0.601	

* Patients were divided according to the use and non-use of lower body perfusion (groups L and NL)

** Bentall = ascending aortic replacement with aortic valve prosthesis

AKI=acute kidney injury; CPB=cardiopulmonary bypass; Cr=creatinine; ICU=intensive care unit; LVEF=left ventricular ejection fraction; SMD=standardized mean difference

**Table 3:** Comparison of perioperative influencing factors after matching.

Indicator	Group L^*^ (n=85)	Group NL^*^ (n=85)	*P*-value	SMD	OR	95% CI
Demographic characteristics						
Male (%)	65 (76.5)	66 (77.6)	1.000	0.028	1.069	0.523-2.185
Age, years (X±s)	52.0±11.0	52.66±11.54	0.694	0.060	1.005	0.979-1.033
Weight (kg)	76.9±14.3	78.0±15.8	0.662	0.067	1.005	0.985-1.025
Preoperative factors						
Smoking history (%)	53 (62.4)	53 (62.4)	1.000	0.001	1.000	0.538-1.860
Diabetes (%)	1 (1.2)	2 (2.4)	1.000	0.089	2.204	0.180-22.752
Hypertension (%)	70 (82.4)	68 (80.0)	0.844	0.060	1.667	0397-1.852
Hyperlipidemia (%)	8 (9.4)	5 (5.9)	0.564	0.133	1.661	0.189-1.920
Preoperative Cr (µmol/L)	66.8 (58.2, 96.1)	71.0 (62.0, 97.3)	0.351	0.001	1.001	0.994-1.008
LVEF (X±s)	57.7±2.7	57.5±3.2	0.868	0.026	1.036	0.855-1.089
Perioperative factors						
Aortic valvuloplasty (%)	37 (43.5)	42 (49.4)	0.538	0.118	1.267	0.6931-2.318
Aortic valve replacement (%)	3 (3.5)	2 (2.4)	1.000	0.070	1.517	0.107-4.045
Bentall^**^ (%)	11 (12.9)	10 (11.8)	1.000	0.036	1.115	0.359-2.239
CPB time (min)	168 (146, 199)	161 (145, 183)	0.231		1.001	0.994-1.004
Circulatory arrest time (min)	6 (5, 10)	30 (14.5, 34.5)	0.000		1.524	1.303-1.783
Cross-clamping time (min)	101 (85, 127)	92 (79.5, 110.0)	0.010		1.012	0.978-0.999
Minimum nasopharyngeal temperature (ºC)	29.4 (28.8, 30.4)	27.2 (26.0, 27.9)	0.000		2.320	0.330-0.563
Perioperative blood transfusion (%)	52 (61.2)	54 (63.5)	0.874		1.105	0.594-2.057
Maximum lactate during CPB (µmol/L)	2.3 (1.9, 3.2)	4.1 (3.5, 5.1)	0.000		2.255	1.691-3.007
Postoperative factors						
Second operation (%)	2 (2.4)	1 (1.2)	1.000		2.024	0.044-2.553
Second intubation (%)	4 (4.7)	0 (0)	0.129			
Drainage volume (mL)	450 (330, 650)	775 (503, 940)	0.000		1.002	1.001-1.003
Ventilator time (h)	32 (19, 53)	22 (18, 45.5)	0.153		1.005	0.989-1.001
ICU time (h)	45 (42, 88)	42 (25, 67)	0.052		1.003	0.993-1.001
Postoperative hospital stay (days)	15 (11, 21)	15 (10, 15)	0.922		1.006	0.988-1.025
Death (%)	7 (8.2)	5 (5.9)	0.765		1.437	0.212-2.288
Postoperative AKI						
AKI (%)	42 (49.4)	47 (55.3)	0.539		1.266	0.693-2.314
Level I AKI (%)	20 (23.5)	32 (37.6)	0.046		1.962	1.008-3.820
Level II AKI (%)	19 (22.4)	11 (12.9)	0.108		1.938	0.229-1.165
Level III AKI (%)	6 (7.1)	4 (4.7)	0.514		1.538	0.177-2.392

* Patients were divided according to the use and non-use of lower body perfusion (groups L and NL)

** Bentall = ascending aortic replacement with aortic valve prosthesis

AKI=acute kidney injury; CI=confidence interval; CPB=cardiopulmonary bypass; Cr=creatinine; ICU=intensive care unit; LVEF=left ventricular ejection fraction; OR=odds ratio; SMD=standardized mean difference

### Comparison Between the Two Groups After Matching

#### Comparison of Intraoperative Indicators Between the Two Groups

All 170 patients underwent total aortic arch replacement combined with frozen elephant trunk implantation. The differences in circulatory arrest time (6 [5, 10] min *vs.* 30 [14.5, 34.5] min, *P*=0.000), cross-clamping time (101 [85, 127] min *vs.* 92 [79.5, 110.0] min, *P*=0.010), minimum nasopharyngeal temperature (29.4 [28.8, 30.4] ºC *vs.* 27.2 [26, 27.9] ºC, *P*=0.000), and maximum lactate during CPB (2.3 [1.9, 3.2] µmol/L *vs.* 4.1 [3.5, 5.1] µmol/L) were statistically significant between group L and group NL. Other indicators revealed no statistical significance.

#### Comparison of Postoperative Indicators Between the Two Groups

There were no significant differences between the two groups in the proportion of the second operation, proportion of second intubation, ventilator time, intensive care unit (ICU) time, postoperative hospital stays, mortality, overall AKI, level II AKI, and level III AKI. Postoperative drainage volume in group L (450 [330, 650] mL *vs.* 775 [503, 940] mL, *P*=0.000) was significantly lower than that in group NL. However, the incidence of level I AKI (20 [23.5] *vs.* 32 [37.6], *P*=0.046) in group L was significantly lower than that in group NL.

## DISCUSSION

Deep hypothermic circulatory arrest (DHCA) was first introduced by Griepp et al.^[9]^ in 1975 and has become the basis of brain protection during aortic arch surgery. The advantage of DHCA is its ability to simply reduce the systemic metabolism level through hypothermia, including that of the brain, thus improving the hypoxic tolerance of brain tissue during ischemia and achieving a good effect in operation with circulatory arrest time < 30 minutes. In order to prolong the safe time limit of aortic arch operation, synchronous cerebral perfusion has gradually become the main brain protection method in hypothermic circulatory arrest^[10]^. At the same time, as the aortic arch operation of the cardiac surgery team gradually gained more maturity, the circulatory arrest management strives to shorten the time of CPB and aortic occlusion as much as possible, thus reducing the requirements for hypothermia^[11]^. In our cardiac surgery center, MoHCA had become the main method in aortic arch surgery, even in minimal invasive incision^[7]^.

LBP is mainly used to provide blood perfusion for distal organs and spinal cord during lower body ischemia and hypoxia. This method has proven effective protection for terminal organs and has already been successfully applied in many aortic arch replacement surgeries^[12]^. Currently, there are three main methods for LBP: balloon occlusion of descending aorta, retrograde perfusion of inferior vena cava, and balloon cannulation^[13-15]^. The balloon occlusion of descending aorta serves to achieve antegrade LBP by occluding the descending aorta and performing femoral artery intubation with an inflatable catheter. In their study, Sun et al.^[16]^ designed and assembled a balloon occlusion device that shortened the circulatory arrest time from 20-25 minutes to five minutes. This method is easy to operate; however, there are risks of false cavity perfusion and embolization. The balloon cannulation method used in this study was based on inserting the balloon intubation into the descending aorta. During the operation, the venous cannula with balloon lumen was inserted into the descending aorta through four branches of artificial vessels, the blood returning was occluded by balloon, and the cannula was intubated to complete antegrade LBP^[4]^. The advantages of this method are physiological antegrade perfusion, control of blood reflux in the surgical field, and no additional incisions. In this study, LBP was carried out in group L, which significantly shortened the circulatory arrest time in group L compared to group NL (6 [5, 10] min *vs.* 30 [14.5, 34.5] min). Because the time of hypoxia shorten significantly, which caused the lactate during CPB in group L to be significantly lower than that in group NL (2.3 [1.9, 3.2] µmol/L *vs.* 4.1 [3.5, 5.1] µmol/L), even the circulatory arrest temperature in group L was significantly higher than that in group NL (29.4 [28.8, 30.4] ºC *vs.* 27.2 [26, 27.9] ºC). These results indicated that LBP can improve organ perfusion and reduce dependence on temperature during circulatory arrest.

CSA-AKI is a recognized complication after cardiac surgery and an important risk factor for death of patients undergoing cardiac surgery. It can prolong ICU and hospitalization times and even increases operative mortality by three to eight times^[17]^. Renal hypoxia during CPB, especially renal medullary ischemia and hypoxia, is an important factor leading to AKI after aortic arch surgery^[18]^. Previous studies have shown that the incidence of AKI in patients with acute type A aortic dissection can range from 20.2 to 66.7%^[6]^. In this study, the overall incidence of AKI in group L and group NL was 49.4% and 55.3%, respectively, which was similar to that in previous studies. Moreover, the incidence of level I AKI (20 [23.5] *vs.* 32 [37.6]) in group L was significantly reduced compared with group NL, which may be related to the significant shortening of ischemia time and anaerobic metabolism time of lower body organs caused by LBP. However, there was no significant difference in levels II and III AKI incidence. Moreover, the incidence of level III AKI in the present study was lower than in the previous study^[6]^, which may be related to the inclusion and exclusion criteria. In addition, although the incidence of postoperative level I AKI in group L was significantly lower than that in group NL, there was no significant difference in ventilator time, ICU stay time, postoperative hospitalization stay, and mortality between the two groups, which may be due to relatively mild kidney injury caused by level I AKI that had limited impact on perioperative clinical outcomes. Long-term follow-up might be good to evaluate the effect of LBP improved prognosis.

### Limitations

This study has some limitations. First, this is a retrospective study that was based on the comparison of time, which may be influenced by time bias. Second, the application of surgical technique was greatly influenced by the surgeon, which may cause selection bias. Third, although this retrospective study used PSM to reduce the selection bias to a certain extent, it was not a prospective controlled study after all. Third, as the LBP flow rate in this study was selected according to clinical experience and pump pressure, it needs to be further investigated. Future studies can avoid these limitations by increasing the sample size and using randomized controlled trials and long-term follow-up.

## CONCLUSION

Antegrade LBP is safe and feasible in aortic arch surgery. It can significantly shorten the circulatory arrest time, making it unnecessary to use DHCA. At the same time, it ensures sufficient oxygen supply to visceral organs during CPB, reducing the incidence of postoperative level I AKI.
